# Smartphone Applications Utilizing Biofeedback Can Aid Stress Reduction

**DOI:** 10.3389/fpsyg.2016.00832

**Published:** 2016-06-17

**Authors:** Alison Dillon, Mark Kelly, Ian H. Robertson, Deirdre A. Robertson

**Affiliations:** Trinity College Dublin, DublinIreland

**Keywords:** stress, biofeedback, technology, skin conductance, electrodermal activity

## Abstract

**Introduction:** Stress is one of the leading global causes of disease and premature mortality. Despite this, interventions aimed at reducing stress have low adherence rates. The proliferation of mobile phone devices along with gaming-style applications allows for a unique opportunity to broaden the reach and appeal of stress-reduction interventions in modern society. We assessed the effectiveness of two smartphone applications games combined with biofeedback in reducing stress.

**Methods:** We compared a control game to gaming-style smartphone applications combined with a skin conductance biofeedback device (the Pip). Fifty participants aged between 18 and 35 completed the Trier Social Stress Test. They were then randomly assigned to the intervention (biofeedback game) or control group (a non-biofeedback game) for thirty minutes. Perceived stress, heart rate and mood were measured before and after participants had played the games.

**Results:** A mixed factorial ANOVA showed a significant interaction between time and game type in predicting perceived stress [*F*(1,48) = 14.19, *p* < 0.001]. Participants in the biofeedback intervention had significantly reduced stress compared to the control group. There was also a significant interaction between time and game in predicting heart rate [*F*(1,48) = 6.41, *p* < 0.05]. Participants in the biofeedback intervention showed significant reductions in heart rate compared to the control group.

**Discussion:** This illustrates the potential for gaming-style smartphone applications combined with biofeedback as stress reduction interventions.

## Introduction

Stress is a major health problem (Kalia, 2002) which is associated with multiple causes of death including heart disease, cancer, and stroke (Cohen et al., 2007) and with most major mental health problems including depression, PTSD and anorexia (Marin et al., 2011). A recent paper from Harvard and Stanford Business Schools on mortality relating to stress, found that problems associated with job stress such as hypertension, cardiovascular disease and decreased mental health lead to 120,000 deaths in America each year (Goh et al., 2015). This makes work related stress and its associated maladies a greater source of morbidity than diabetes, Alzheimer’s Disease or influenza. Additionally the study found that stress-related problems could be responsible for between 5 and 8% of annual healthcare costs in the US amounting to about $180 billion per annum.

A number of effective therapeutic interventions for stress have been developed (Carson and Kuipers, 1998; Doherty et al., 2012). However, often interventions suffer several challenges related to delivery, namely low adherence rates and low engagement rates (Paredes et al., 2014). One type of intervention uses biofeedback as an aid to stress reduction.

Biofeedback detects and presents physiological signals such as heart rate, respiration, muscle activity, or skin temperature from the user’s body, and by making users aware of these signals, helps them to gain control over them (Schwartz et al., 2008; Micoulaud-Franchi et al., 2014). The concept has demonstrated value for stress reduction in studies on stress management for hospital nurses (Cutshall et al., 2011) and for veterans suffering with Post Traumatic Stress Disorder after 9/11 (Reyes, 2014). Biofeedback is a component of a number of stress reduction programs (e.g., improving stress management skills in soldiers (Bouchard et al., 2012) and reducing stress in pregnant women (Keeney, 2008) because it teaches users to recognize when they are stressed from their own physiological measurements, and teaches them how to control both their psychological stress levels and the physical symptoms of stress.

Many of these stress programs, however, teach participants to regulate their stress response in quiet, peaceful surroundings and in unchallenging conditions, something that may not transfer easily to more stressful conditions where the skill is really needed. Modern biofeedback devices are thus often combined with video games that contain a competitive element. They teach individuals to try to control stress in more complex and less controlled, competitive and distracting environments. For example, Vilozni et al. (2001) developed a competitive videogame that taught breathing skills to children. The game operated via a positive feedback loop, whereby children learned deep breathing skills by observing an animated character on a computer screen who progressively moved as the children took deeper breaths (Vilozni et al., 2001). Similarly Bouchard et al. (2012) found that soldiers who received three sessions of biofeedback assisted stress management training while immersed in a competitive shooter game were more effective in controlling stress (as measured by cortisol response and heart rate) in a subsequent stress induction task, compared to a control group who received no stress management training. In relation to stress management for health, a study conducted by Leahy et al. (1997) found that patients with irritable bowel syndrome, a condition to which stress is a major contributor, had reduced global and bowel symptoms scores after four 30 min biofeedback sessions on a competitive computer game (Leahy et al., 1997). Research of this nature opens many avenues for the combination of emerging technologies with age-old stress reduction techniques. However, most of this research has been conducted in the laboratory or using desktop computers that mean users have to be in one place to avail of the interventions.

The proliferation of smartphone devices offers a new platform for delivering mobile stress management programs that users can engage with in any setting. As summarized by Preziosa et al. (2009), mobile phones can respond to different clinical needs (Preziosa et al., 2009). Firstly, the diffusion of mobile phones reduces the digital divide and guarantees the availability of the contents anytime and anywhere. According to a recent study by 90% of the world’s population over the age of six will have a mobile phone by the year 2020 (Ericsson Ltd., 2014). Secondly smartphones can provide brief and semi structured interventions aimed at helping individuals to manage their emotions. Finally, smartphones are equipped with several sensing capabilities which permit the detection, recognition, and identification of a number of activities and context information which can be converted into reports of personal health data (Preziosa et al., 2009).

The advantages of mobile phones for health interventions have been shown in a number of studies. One study used mobile phone applications to provide stress inoculation training to oncology nurses (Villani et al., 2013). Another recent study successfully taught individuals relaxation exercises via a competitive respiratory biofeedback game (Parnandi et al., 2014), and another used a heart rate biosensor device connected to a free positive technology app to successfully teach breathing exercises and thereby reduce levels of arousal (Serino et al., 2014).

The utility of biofeedback has been demonstrated in a number of forms, most notably by Galvanic Skin Response (GSR) devices, which has proven to be a reliable method of measuring activity of the sympathetic medullary system. This system is responsible for the secretion of adrenaline and noradrenaline during the stress response (Graeff et al., 2003; El-Sheikh et al., 2008; Allen et al., 2014). GSR measures the electrical conductance of the skin. When the sympathetic branch of the autonomic nervous system is highly aroused, sweat gland activity also increases, which in turn increases skin conductance (Graeff et al., 2003). Previous studies have used skin conductance as the physiological marker for biofeedback interventions (e.g., Leahy et al., 1997).

The aim of this study was to test the efficacy of a biofeedback stress management intervention which combines a mobile smartphone device and gaming style apps. The biofeedback device used in this study was the Pip (Galvanic Ltd, Ireland) that captures changes in skin conductivity. This information is transmitted via Bluetooth to smartphone applications, where algorithms analyze the electrodermal activity and use it to determine progress in the games. The more relaxed the player, the greater the progress in the game. This study aimed to investigate whether two smartphone applications, ‘The Loom’ and ‘Relax and Race,’ effectively reduced self-reported measures of stress and physiological measures of stress compared to a control game, ‘Flow Free.’ In conjunction with this aim, changes in self-reported measures of mood were also under investigation. We hypothesized that the biofeedback related game would cause greater reductions in psychological and physiological levels of stress after a stress induction compared to a control game.

## Materials and Methods

### Participants

Fifty healthy adults participated in the study (32 female, 18 male, age: *M* = 26.7, *SD* = 5.1). Participants were randomly allocated to the experimental biofeedback (B) or the control condition (C). There were 25 participants in each condition and participation lasted 60 min. The Beck Anxiety Inventory (Steer and Beck, 1997) was initially completed to determine if there were any underlying anxiety problems, which would exclude participation in the study. Participants with a score of 36 or above were to be excluded, however, this did not apply to any participants in the sample. Study protocols were all in accordance with the declaration of Helsinki and were approved by the Ethics Committee of Trinity College, Dublin.

### Design

The study was an independent groups design with repeated measures over a time of 30 min. Participants in the biofeedback condition played the mobile phone application games ‘Relax and Race’ and ‘The Loom’ (Galvanic Ltd, 2012). Participants in the control condition played the mobile phone application game ‘Flow Free’ (Big Duck Games, 2012; see details below). Participants’ heart rates, mood and perceived stress levels were recorded before and after playing the games. We hypothesized that participants in the biofeedback condition would have a greater decrease in heart rate, perceived stress levels and improved mood after playing the intervention games compared to the control condition.

### Measures

#### Anxiety

Participants completed the Beck Anxiety Inventory (Steer and Beck, 1997). This consisted of a list of 21 symptoms such as ‘fear of worst happening’ and ‘hands trembling.’ Participants were asked to rate how much they had been bothered by each symptom over the past month on a 4-point scale of 0 to 3 (‘not at all’ to ‘severely’). The measure has been shown to have good reliability and validity (Steer and Beck, 1997). The Anxiety Inventory was used to exclude participants with potential clinical levels of anxiety as evidenced by a score greater than 36. No participants were excluded on this basis.

#### Mood

The UWIST Mood Adjective Checklist (UMACL; Matthews et al., 1990) was used to analyze participants’ mood. The UMACL consists of a list of 34 adjectives rated on a 4-point response scale; participants judge the extent to which these terms describe their current mood on a scale ranging from ‘definitely’ to ‘definitely not’ and circle the number beside the adjective that best describes their mood at the present time. This instrument was designed to model the entire affective space and includes adjectives to measure the affective dimensions of Tense Arousal (Cronbach’s alpha for this sample = 0.78), Hedonic Tone (α = 0.74) and Energetic Arousal (α = 0.42; Matthews et al., 1990).

#### Stress Level

A Visual Analogue Scale (VAS; Lesage et al., 2012) was used to determine participants’ level of stress. Participants were asked to indicate how stressed they currently felt with 1 being not at all and 10 being the maximum possible feeling of being stressed.

#### Heart Rate

An iHealth^®^ wireless pulse oximeter was used to measure heart rate through participants’ right index finger (iHealth Lab Inc., 2012).

#### Galvanic Skin Response

The Pip (Pip^®^, Galvanic Ltd) was used to measure skin conductance. The Pip is a wireless biofeedback device that measures electrodermal activity (EDA). The sensors detect EDA in the player’s fingertips eight times per second. This is transmitted via Bluetooth to the Pip’s Apps whereby a propriety algorithm analyses changes in EDA and uses this to determine progress in the apps.

An Apple iPhone 4S 16GB mobile phone with the games; the Loom, Relax and Race, and Flow Free installed was used by all participants.

#### Trier Social Stress Test

All participants completed the Trier Social Stress Test (TSST; Kirschbaum et al., 1993) in order to induce moderate psychological stress. This consisted of three stages. The first was the anticipatory stress phase during which participants were brought into a room at which two people were already sitting at a table. The participant was asked to stand in front of them and was given the following instructions: *“You must imagine you are applying for you dream job. You have dreamt about working in this job for as many years as you can remember. After seeing an advertisement for this perfect job, you have decided to apply. After submitting the application you have been invited for an interview. The salary for this job is very high, there is a lot of competition for the job, and the final selection will be made based on your ability to convince us, the interviewers of how your experiences, abilities and education make you a better candidate than anyone else. You will have three minutes to prepare a detailed speech explaining why you are the ideal candidate for this job and you will be given a sheet of A4 paper to make notes on.”* After the 3 min preparation time had elapsed, one of the interviewers took away the A4 sheet with the participant’s notes on it and the second phase began. The participant had to deliver a 5-min speech explaining why he or was the ideal candidate for the job. Throughout this the interviewers maintained a neutral expression and observed the participant without comment. The third phase was the mental arithmetic task in which participants were asked to count backward from 1,022 in steps of 13 as fast and as accurately as possible. If the participants made a mistake, they were asked to start again from the beginning. This phase lasted for 5 min. The TSST has been shown to be a valid and reliable instrument for inducing physiological and psychological stress responses in people of all ages (Hellhammer and Schubert, 2012). In a recent meta-analyses of 165 laboratory stress studies, the TSST was found to produce the most robust physiological stress responses as compared with several other stress tasks (Dickerson and Kemeny, 2004).

#### Interventions

The first intervention game, ‘Relax and Race’ is a competitive racing game in which the player is represented by a small green dragon. As the player relaxes and their skin conductance decreases, as measured by the Pip, the dragon flies higher and faster. Should the player become stressed, and therefore have an increase in skin conductance, this causes the dragon to slow down. Therefore, the player who relaxes most wins the race. In the single player version – used in this study – the player competes against a ‘ghost dragon’ representing his most recent best score so that he can compete against himself’ and improve his relaxation over time. Winning the race provides a motivating reward for achieving a relaxed state.

The second intervention game, ‘The Loom’ is a single player game, which commences with a visual image of a frozen snow covered scene. The objective of the game is to progress from the winter scene through to a summer scene. The more relaxed the player is, the faster the transition. As the player relaxes and their skin conductance decreases, the landscape responds.

The control game, ‘Flow Free’ is a single player puzzle game with pairs of matching colors dotted in different locations across a board. The objective of the game is to connect matching color pairs with a pipe in order to create a flow. The puzzle is solved once each pair has been matched and the entire board is covered. If overlap of piping occurs, however, the pipes break and the game is lost. There are 30 different levels of ‘Flow Free,’ level one is the most basic level and is very simple and relaxed, level thirty is the highest level and is very complex and frenetic. Flow Free was chosen as the control game as it is a stimulating game, yet does not induce high levels of stress when the player begins playing at a low level and progresses to higher levels at their own leisure and according to their own abilities.

### Procedure

Study participants were given an information sheet with study information and a signed consent form. All participants were tested individually between the times of 10am and 4pm, participation lasted one hour. On arrival, participants rested for 10 min in Room A. After 10 min they were taken to a second room (Room B) in which the TSST would take place (see “Materials and Methods”). After completing the TSST, participants’ heart rates were taken using the pulse oximeter, which required the participant to place his/her right index finger into plastic cuff for approximately 20 s until a heart rate measure could be read. Participants then completed the perceived stress and mood measures. Following this participants were randomly selected to take part in the biofeedback or the control conditions. Participants in the biofeedback condition held the Pip between their index finger and thumb. All participants were given instructions on how to play the games. The participants who played the games ‘The Loom’ and ‘Relax and Race’ played each of the games for 15 min. Participants who played the control game, ‘Free Flow’ started on the easiest level worked their way up to more difficult levels at their own pace. Each participant played the smartphone application games for 30 min. After participants had played the games for 30 min, heart rate measures, perceived mood and stress levels were recorded again. Participants were then debriefed and informed of the purpose of the study.

## Results

### Self-reported Levels of Stress

The means and standard deviations for all treatment conditions are shown in **Table 1**. All assumptions were met and therefore it was appropriate to continue with a mixed factorial ANOVA. A 2 × 2 mixed factorial ANOVA showed a significant main effect of time, *F*(1,48) = 60.89, *p* < 0.001, $\eta_{p}^{2}$ = 0.56, such that there was a significant reduction in stress levels over time, as measured from the pre-gaming time (*M* = 5.01, *SD* = 1.94) to post gaming (*M* = 3.27, *SD* = 1.97). There was no significant main effect of game *F*(1,48) = 2.07, *p* > 0.05.

**Table 1:** Mean heart rate and stress levels for participants in the biofeedback and control groups before and after they completed the games.

Variable (Possible Range)	Biofeedback Condition Mean (*SD*)	Control Condition Mean (*SD*)
Perceived Stress Before	5.08 (2.05)	4.94 (1.86)
Perceived Stress After	2.5 (1.68)	4.04 (1.97)
Heart Rate Before	78.64 (17.08)	81.08(18.13)
Heart Rate After	71.84 (13.82)	79.64 (17.89)
Hedonic Tone Before	14.84 (3.30)	15.84 (3.16)
Hedonic Tone After	14.72 (1.62)	15.60 (2.38)
Tense Arousal Before	26.32 (2.67)	24.24 (3.80)
Tense Arousal After	27.68 (3.44)	25.36 (4.11)
Energetic Arousal Before	26.92 (3.34)	23.20 (3.15)
Energetic Arousal After	27.52 (3.90)	23.52 (4.15)

**FIGURE 1:**
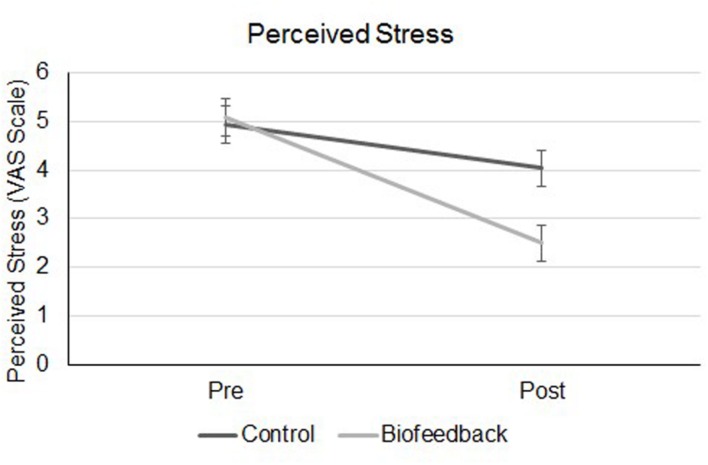
**Perceived stress measurements for participants in the biofeedback and control groups, before and after they completed the games**.

There was a significant interaction between time and game type, *F*(1,48) = 14.19, *p* < 0.001, $\eta_{p}^{2}$ = 0.23 (see **Figure 1**). Paired sample *t*-tests were used as *post hoc* analysis to identify the source of the interaction. Participants who played the biofeedback games had a statistically significant decrease in stress levels [Mean Before = 5.08 (*SD* = 2.05); Mean After = 2.5 (*SD* = 1.68), *t*(24) = 7.48, *p* < 0.001, two-tailed]. Participants who played the control application game, also had a reduction in stress levels but to a lesser amount [Mean Before = 4.94 (*SD* = 1.86), Mean After = 4.04 (*SD* = 1.97), *t*(24) = 3.18, *p* < 0.01, two-tailed]. These results indicate that the biofeedback applications ‘Relax and Race’ and ‘The Loom’ were more effective at reducing self-reported levels of stress in participants compared to the ‘Flow Free’ game.

### Mood Analysis

The UWIST Mood Adjective Scale was analyzed from the perspective of three separate dimensions: hedonic tone, tense arousal and energetic arousal. Three mixed factorial ANOVAs were conducted to determine if there was a change in self-reported mood levels before and after the stress reduction period. The means and standard deviations for all treatment conditions are shown in **Table 1**.

There was no interaction effect between time and condition in predicting hedonic tone [*F*(1,48) = 0.02, *p* > 0.05], tense arousal [*F*(1,48) = 0.05, *p* > 0.05] or energetic arousal [*F*(1,48) = 0.08, *p* > 0.05]. These results indicate that there was no significant change in mood following the stress reduction intervention.

### Heart Rate

A 2 × 2 mixed factorial ANOVA showed a significant main effect of time for heart rate, *F*(1,48) = 15.14, *p* < 0.001, $\eta_{p}^{2}$ = 0.24, such that there was a significant reduction in heart rate over time, as measured from the pre-gaming time (*M* = 79.86, *SD* = 17.48) to post gaming (*M* = 75.74, *SD* = 16.30).

**FIGURE 2:**
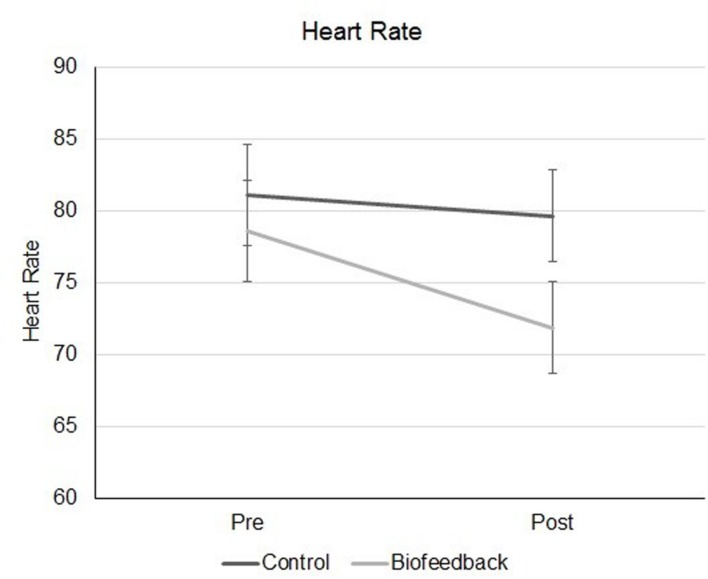
**Heart rate measurements for participants in the biofeedback and control groups, before and after they completed the games**.

There was no significant main effect for game, *F*(1,48) = 1.22, *p* > 0.05, $\eta_{p}^{2}$ = 0.03. There was a significant interaction between time and game, *F*(1,48) = 6.41, *p* < 0.05, $\eta_{p}^{2}$ = 0.12 (see **Figure 2**).

Paired sample *t*-tests were used as a *post hoc* analysis to identify the source of the interaction. Participants who played the biofeedback games had a statistically significant decrease in heart rate [Mean Before = 78.64 (*SD* = 17.08), Mean After = 71.84 (*SD* = 13.82), *t*(24) = 4.41, *p* < 0.001, two-tailed]. Participants in the control condition did not have a statistically significant reduction in heart rate [Mean Before = 81.08 (18.13), Mean After = 79.64 (*SD* = 17.89), *t*(24) = 0.99, *p* > 0.05].

## Discussion

In the present study, we analyzed the effectiveness of smartphone application games combined with biofeedback in the reduction of physiological and psychological markers of stress. We found that 30 min of biofeedback training significantly reduced self-rated stress and heart rate in a group of temporarily stressed participants compared to a control procedure. Specifically smartphone games ‘Relax and Race’ and ‘The Loom,’ played via an electrodermal sensor (the Pip), for 15 min each, reduced self-reported stress following a stress-induction procedure by 50% compared to 18% in a control group and heart rate by 8% compared to 2% in a control group. This significant reduction suggests that smartphone application biofeedback games may be an effective method to teach users to manage their stress. Although this was only a short intervention the efficacy of a smartphone and mobile biofeedback device in reducing physiological and psychological markers of stress illustrates the potential of scalability of these types of interventions. Gaming-style biofeedback applications on smart devices that can be, and are, carried anywhere may broaden the reach and appeal of stress management interventions thereby increasing adherence and efficacy.

One key aspect of biofeedback is that it gives users a tangible external measure of their stress response. As they are able to monitor their stress responses they then learn to control them more effectively. The resulting perceived control over stress could be a vital cognitive outcome of the game, as it has often been stated that stress is induced due to a perceived lack of control over a particular situation or stimulus (Eysenck, 2013). Leotti et al. (2010) showed that perceived control over a situation or stimulus can inhibit autonomic arousal. Also of note is the fact that removing control can produce heightened sensitivity toward a stressor, resulting in increases in rumination and negative thought patterns. Wallston et al. (1994) showed that a person’s ‘locus of control’ or the perception that they have control over their own health can affect the health of the individual.

Using competitive games as a means to teach relaxation techniques through biofeedback may lead to better transfer of relaxation skills to other stressful tasks in the real world. This was evident in prior research on stress exposure in military settings (Bouchard et al., 2012) which showed that for many tasks normal training procedures do not improve performance when the task is later to be performed under stress. Combining multiple-parameter biofeedback with training, however, resulted in improved stress management in simulated situations indicating that the skills learnt could be effectively carried over. Similarly, Parnandi et al. (2014) compared a competitive respiratory biofeedback smartphone game to a conventional non-biofeedback version of the game and to traditional relaxation based on deep breathing. They found that heart rate levels for participants in the biofeedback gaming condition reduced significantly from pre-gaming to post-gaming compared to those in the other two conditions indicating successful transfer of relaxation skills.

Lichstein (1988) outlines a number of meditative practices and relaxation techniques that may help to prevent stress from building up to unmanageable levels. Within these practices runs the theme of trying to become aware of unconscious bodily processes. The skin conductance response sensor utilized with ‘Relax and Race’ and ‘The Loom’ is capable of measuring very minute changes in physiological processes, making it possible for users to become aware of these unconscious bodily responses. Given the nature of today’s mobile phone centric society, engaging games utilized with biofeedback is a very practical means of helping individuals reduce their stress levels in a number of settings. Allen and Blanchard (1980) provided evidence that biofeedback was capable of reducing levels of stress in business managers. Since then, biofeedback has developed in terms of its practicality and is much more accessible to the public due to the advances in mobile phone technology.

Most studies, including our own, investigated only short-term interventions using biofeedback and gaming interventions. There is, however, some evidence that these types of interventions can be used for longer term outcomes. Yahav and Cohen (2008) found that an 8-week long intervention for adolescents involving biofeedback combined with a game reduced state anxiety and improved self-esteem. Similarly, Leahy et al. (1997) found that patients with Irritable Bowel Syndrome continued to use a computerized biofeedback game to manage symptoms at long-term follow up after the study despite there being no further contact with the hospital where the study was carried out. More studies are needed to assess the long term efficacy of these types of interventions but these studies indicate the feasibility of game-based biofeedback as a long-term intervention. Similarly, the aim of this study and many others is to assess the efficacy of a game-based biofeedback intervention for stress in a non-clinical sample. There is some indication, however, that game-based biofeedback could be useful for clinical samples as well. Knox et al. (2011) found that an 8-week intervention using game-based biofeedback was successful in reducing symptoms of anxiety and depression in a group of 9- to 17-year-olds presenting with clinically relevant symptoms compared to waiting list controls. More studies in this area are needed before recommending interventions for this and other clinical populations but in future it may be possible to use game-based biofeedback as an adjunct therapy for those on waiting lists or between psychological sessions.

The study indicates the potential efficacy of biofeedback smartphone applications as stress reduction tools. There are, however, a number of limitations to this study. Firstly, although we found a statistically significant difference the size of the effect was relatively small. Secondly, we could not distinguish between the efficacy of the Relax and Race and Loom games and it may be that one was more effective than the other. We chose to use two games in the biofeedback condition to avoid the negative effects of boredom which may have confounded results had we only included one. The control game moved through different levels as participants progressed and thus using two biofeedback games (that did not progress through levels) made this condition more similar to the control game. However, the control and biofeedback interventions did use different games leading to the question as to whether group differences may have arisen because of differences in the games rather than biofeedback. We attempted to control for this by comparing games which were presented on the same medium (i.e., a smartphone) and both of which had a competitive element involving beating a personal best. The control game was chosen to control for the possibility that mere distraction from a stressful task through gaming would reduce stress. However, only participants playing the biofeedback-related games showed a reduction in both heart rate and perceived stress following the stressful task indicating that distraction was not the driving factor in stress reduction.

A further point to note is that we did not take measurements of heart rate at baseline (prior to the TSST) meaning we do not know what participants’ normal heart rate levels are. However, our aim in this study was to create an equivalent state of stress in the two groups and compare change from this stress-state baseline to post-intervention. As can be seen by the mean level of heart rate in the two groups following the TSST [Biofeedback = 78.64 (17.08); Control = 81.08 (18.13)] we achieved similar levels of stress in the two groups.

Finally, the internal reliability of the energetic arousal subscale was low (α = 0.42). It is not clear why this was the case, however, we did not have a directional hypothesis for this specific subscale and did not find significant changes for the two mood subscales that did show good reliability suggesting that it is unlikely the low reliability for this one subscale significantly affects the overall study findings.

## Conclusion

Two 15-min biofeedback-controlled smartphone games produced significant short-term reductions in psychological and physiological measures of stress, compared to a control smartphone game. This study complements other research and highlights the fact that biofeedback enabled applications offer a potential solution to the demand for efficient, low cost, and stigma-reducing interventions in the treatment of stress (Litz and Salters-Pedneault, 2008). Future research may look to test the applications outside of the laboratory environment and for a longer period of time as well as in a clinical population.

## Author Contributions

AD, MK, and IR designed study. AD, MK, IR, and DR were involved in interpretation of results. AD and MK collected and analyzed data. AD, MK, IR, and DR were involved in write-up and review.

## Conflict of Interest Statement

IR is the Chair of the Scientific Advisory Board of Galvanic Ltd. DR sits on the Scientific Advisory Board of Galvanic Ltd and acts as a paid consultant for the company. The other authors declare that the research was conducted in the absence of any commercial or financial relationships that could be construed as a potential conflict of interest.
